# Valorization of Tomato Leaves: Optimization of Eco-Friendly Phenolic Extraction and Assessment of Biological Activities

**DOI:** 10.3390/foods14193383

**Published:** 2025-09-30

**Authors:** Layan Helmi, Suhair Sunoqrot, Samah Abusulieh, Rawan Huwaitat, Espérance Debs, Salma Khazaal, Mohammad H. El-Dakdouki, Nicolas Louka, Nada El Darra

**Affiliations:** 1 Department of Biological Sciences, Faculty of Science, Beirut Arab University, Beirut 1107 2809, Lebanon; l.ahelmi@bau.edu.lb; 2 Department of Pharmacy, Faculty of Pharmacy, Al-Zaytoonah University of Jordan, Amman 11733, Jordan; suhair.sunoqrot@zuj.edu.jo (S.S.); samahmaher996@gmail.com (S.A.); r.huwaitat@zuj.edu.jo (R.H.); 3 Department of Biology, Faculty of Arts and Sciences, University of Balamand, Tripoli 1300, Lebanon; esperance.debs@balamand.edu.lb; 4 Department of Nutrition and Dietetics, Faculty of Health Sciences, Beirut Arab University, Tarik El Jedidah, Riad EL Solh, Beirut 1107 2809, Lebanon; s.khazaal@bau.edu.lb; 5 Department of Chemistry, Faculty of Science, Beirut Arab University, Riad El Solh, Beirut 1107 2809, Lebanon; m.eldakdouki@bau.edu.lb; 6 Centre d’Analyses et de Recherche, Unité de Recherche Technologies et Valorisation Agro-Alimentaire, Faculté des Sciences, Université Saint-Joseph de Beyrouth, Mar Roukos, Dekwaneh, Riad El Solh, Beirut 1107 2050, Lebanon; nicolas.louka@usj.edu.lb

**Keywords:** tomato leaves, LC-MS, polyphenols, antioxidant, anti-cancer, anti-inflammatory, agricultural waste management

## Abstract

Tomato leaves, typically discarded during harvest, are a rich yet underutilized source of bioactive compounds. This study aimed to valorize tomato leaves by optimizing the extraction of their phenolic compounds using a water-based method and response surface methodology. The optimal conditions, notably heating a mixture of 1:50 solid-to-liquid ratio at 71 °C for 29 min, yielded the most total phenolic content and antioxidant activity. The biological activities of the lyophilized tomato leaf extract (TLE) were then assessed. TLE showed dose-dependent antimicrobial activity against *Escherichia coli* and *Candida albicans*, but neither against *Pseudomonas aeruginosa* nor *Staphylococcus aureus*. In addition, it demonstrated moderate cytotoxicity against MCF-7 breast cancer cells with an IC_50_ value of 114.5 µg/mL. Interestingly, the extract significantly reduced intracellular reactive oxygen species levels in RAW 264.7 macrophages, supporting its anti-inflammatory potential. LC-MS analysis identified rutin (45.21%), 4-hydroxycoumarin (13.60%), and α-tomatine (12.37%) as the major chemical constituents in TLE, suggesting contributing effects behind the observed bioactivities. These results support the potential of tomato leaf extract as an eco-friendly source for functional ingredients, transforming agricultural waste through green extraction into valuable applications for nutraceuticals and sustainable product development.

## 1. Introduction

Tomatoes (*Lycopersicon esculentum* Mill.) belong to the *Solanaceae* family and are one of the most widely consumed vegetables worldwide. In 2022, the annual production of tomatoes was estimated at 186.82 million tons covering a total area of 5 million hectares with an average productivity of 36.97 tons/ha [1]. Tomato leaves represent an agricultural residue from tomato cultivation with more than 120 million tons have been generated annually over the past decade [2]. These leaves are generally discarded along with the stems, resulting in significant waste whose improper disposal can lead to detrimental environmental impacts, including increased greenhouse gas emissions, soil degradation, and water pollution. However, the growing quest for sustainability in food and agriculture sectors is driving research towards innovative strategies to valorize and repurpose agricultural by-products.

Tomato leaves contain alkaloids, flavonoids, and phenolic compounds with valuable biological benefits and industrial potential. Phenolics specifically are important secondary metabolites although they usually constitute less than 1% of the plant’s dry weight, [3,4]. They have a role in defending plants against environmental stressors like heat, UV radiation, pests, and pathogens [5]. Flavonoids have been shown to protect plants from UV-B radiation, increasing with light exposure and degrading in darkness [6]. Moreover, Arab et al. evaluated the levels of phenolic compounds in the methanolic extracts of the leaves of eight varieties of tomato and reported total phenolic contents ranging between 162 and 240 mg gallic acid equivalent (GAE) g^−1^ DW [7].

Silva-Beltrán et al. evaluated the individual phenolic compounds present in tomato leaf extract (TLE), using an ethanolic–acetic acid mixture as the extracting solvent and HPLC to measure the bioactive compounds present. The authors reported that quercetin was the most abundant phenolic compound, suggesting its contribution to the bioactive properties of the extract [8]. Other phenolic compounds detected in the extract included gallic acid, ferulic acid, and chlorogenic acid. Furthermore, caffeic acid and rutin were detected, albeit at relatively low concentrations.

The richness of TLE in bioactive compounds endowed them with notable biological activities and rendered them a valuable sustainable resource for applications such as antimicrobial, antioxidant, anti-inflammatory, and anticancer agents. For example, extracts of tomato leaves were found to possess antimicrobial activity. A study by Silva-Beltrán et al. evaluated the growth inhibition potential of tomato leaf, stem, and root extracts against *Escherichia coli* O157:H7, *Staphylococcus aureus*, *Salmonella typhimurium*, and *Listeria ivanovii* [8]. The findings revealed that the leaves exhibited the highest antimicrobial effect against all tested microbial strains. Another study found that acetone extract from tomato leaves inhibited mycelial growth of *Fusarium oxysporum* f. sp. *lycopersici*, *Glomerella cingulata*, and *Rhizoctonia solani* [9]. These results show that tomato leaf extract has antifungal activity in addition to the previously mentioned antibacterial effects, highlighting its potential as an antimicrobial agent.

The anticancer activity of TLE was validated in several reports. In one study, the effect was credited to the steroidal alkaloid tomatidine, which suppressed tumor development in vivo and the reduced growth of human gastric cancer 85As2 cells in vitro [10]. Microarray analysis of gene expressions in tumors collected from mice that were fed the tomatidine rich extract showed changes in the expression levels of mRNAs associated with type I interferon signaling pathway. Furthermore, knockdown of α-inducible protein 27 (*IFI27*), a type I interferon-stimulated gene (ISG), suppressed the proliferation of cultured 85As2 cells. Another study assessed the effect of various tomato leaf extracts and their toxic effects on human gastric adenocarcinoma cells [11]. Acetone, hydromethanol, and alkaloid-purified extracts of tomato leaves reduced cell viability, with the latter having the strongest cytotoxic effect with IC_50_ ranging from 9 ± 2 to 55 ± 11 μg/mL for all selected cultivars. Additionally, the high scavenging activity of hydro methanol extracts, particularly from Anairis (IC_50_ = 0.87 ± 0.12 mg/mL) and Abuela varieties (IC_50_ = 0.89 ± 0.02 mg/mL), against nitric oxide, and their superior performance against superoxide radicals (IC_50_ range: 0.12 ± ≤0.01 to 0.43 ± 0.08 mg/mL), highlights their strong antioxidant potential. These values not only reflected dose-dependent efficacy but also surpassed that of the standard quercetin-3-O-rutinoside in scavenging O_2_•^−^ (IC_50_ = 0.58 mg/mL), suggesting their relevance as natural antioxidant sources.

Based on previous findings, the purpose of this research is to extract bioactive compounds from tomato leaves using water bath extraction (WBE) with distilled water as a green and food safe extracting solvent. Unlike prior studies that relied on organic solvents or sophisticated extraction methods, this method prioritizes safety simplicity, and environmental compatibility. The extraction procedure was optimized using response surface methodology (RSM). To our knowledge, this is the first study to apply WBE using distilled water for tomato leaves in combination with RSM to optimize extraction conditions. Following extraction, the resulting extract was evaluated for its chemical composition, and its antioxidant, antiradical, antimicrobial, anticancer, and anti-inflammatory activities.

## 2. Materials and Methods

### 2.1. Raw Materials

The leaves of tomato plants were collected in August 2023 from the Beqaa Region in Eastern Lebanon, originating from the Ammani plum tomato variety. They were collected after harvesting of the organic tomato fruit from the field. They were washed twice with distilled water to remove unwanted substances and dried indoors for 48 h under natural sunlight exposure through windows. They were then ground using an electric household-level grinder into fine powder with particle size range of 0.5–1 mm and then stored in the fridge at a temperature of 5 °C until ready to be used.

### 2.2. Chemicals and Reagents

The chemicals, obtained in analytical grade, were used without modification in their supplied form. Sodium bicarbonate, 2,2-diphenyl-1-picrylhydrazyl (DPPH), gallic acid, and Trolox were purchased from Sigma Aldrich (St Louis, MO, USA). Folin–Ciocalteu’s phenol reagent was obtained from Oxford Lab Chem (Mumbai, India). Ultrapure water (specific resistivity ~18.2 MΩ.cm at 25 °C) was prepared using a Milli-Q purification system (EMD Millipore, Billerica, MA, USA). Absolute ethanol was procured from Fisher Scientific (Loughborough, UK). Mueller Hinton Agar (MHA) was obtained from Biolab (Budapest, Hungary). Sabouraud Dextrose Agar (SDA) was purchased from Oxoid (Basingstoke, UK). All standards used for LC-MS analysis were obtained from Sigma-Aldrich (St Louis, MO, USA).

### 2.3. Dry Matter Content

The dry matter (DM) was obtained by using a precisely measured 5 g of the leaves and drying in a hot air oven at 105 °C for 24 h then recording the weight loss [12]. The DM of the initial raw material was 91 ± 0.3%.

### 2.4. Preparation of the Tomato Leaves Extract

The tomato leaves powder was extracted using ultrapure water at various solid-to-liquid ratios and temperatures. A defined amount of tomato leaves powder (0.5 g) was placed in a 50 mL Erlenmeyer flask and combined with various amounts of ultrapure water then covered with aluminum foil and submerged in the water bath at different temperatures and duration without stirring. Then, the mix was filtered through No. 1 Whatman filter paper and left to cool to room temperature. The extract was subsequently centrifuged at 4500× *g* for 10 min (Hermle Z326K centrifuge, Gosheim, Germany), and the supernatant was collected for measurement of total phenolic content (TPC), which was used as the basis for determining the best solid-to-liquid ratio to optimize the extraction temperature and time through experimental design.

### 2.5. Experimental Design for Optimization of Tomato Leaves Extraction

The extraction process was optimized using RSM with a central composite design to evaluate the individual and interactive effects of the temperature (T) and time (t) on TPC and antioxidant activity. The design included 12 runs with four replicates at the central point. Temperature ranged from 40 to 80 °C, and extraction time from 15 to 45 min, with the highest and lowest values coded as +1 and −1, respectively.

Based on the two independent variables (temperature and time) and one response (TPC), the data were fitted to a second-order regression model:

Y = α_0_ + α_1_ × T + α_2_ × t + α_3_ × T^2^ + α_4_ × T × t + α_5_ × t^2^

In this model, “Y” indicates the predicted response; α_0_ represents the mean response at the center of the experimental design, α_1_ and α_2_ are the linear coefficients, α_3_ and α_5_ represent the quadratic terms, and α_4_ is the coefficient associated with the interaction between temperature and time.

### 2.6. Determination of Total Phenolic Content

The TPC of each extract was evaluated using the Folin–Ciocalteu method [13,14]. Briefly, 250 µL of gallic acid standard or freshly prepared extract sample were mixed with 1 mL of Folin–Ciocalteu reagent (diluted 1/10, *v*/*v* in ultrapure water). After 5 min, 1 mL of sodium bicarbonate solution (10%) was added, vortexed, and incubated at room temperature in the dark for 30 min. Absorbance was then measured using a UV-Vis spectrophotometer (Specord 250 Plus, Analytik Jena, Jena, Germany) at 765 nm. For the blank, ultrapure water was used instead of the sample, following the same procedure. A gallic acid (GA) calibration curve was prepared to extrapolate TPC, reported as mg GA equivalents per gram of dry matter (mg GAE/g DM). All measurements were carried out in triplicate, and data was reported as the mean ± standard deviation (SD).

### 2.7. Antioxidant Activity Determination

The antioxidant potential of the TLE was performed by measuring its radical scavenging effect on DPPH (2,2-diphenyl-1-picrylhydrazyl) free radical [15]. In this assay, 0.2 mL of each extract, Trolox standard (as positive control), or ultrapure water (as negative control) was mixed with 4 mL of DPPH solution (0.1 mM in ethanol). Following 30 min incubation at room temperature, the absorbance of the samples was measured at 517 nm, using a blank consisting of 4 mL ethanol and 0.1 mL of ultrapure water. Antioxidant capacity was calculated as the percentage of DPPH inhibition using the formula below:

$$Inhibition\;percentage=\;\frac{Absorbance\;of\;the\;Negative\;Control-Absorbance\;of\;the\;Extract}{Absorbance\;of\;the\;Negative\;Control}\;\times \;100\;$$

### 2.8. Freeze-Drying

After optimizing the extraction conditions, TLE was freeze-dried for long term storage and further analysis. Briefly, the liquid sample was stored at −80 °C overnight in a 50 mL plastic vial and then freeze-dried using a FreeZone 4.5 L Benchtop Freeze Dryer (Labconco, Kansas City, MO, USA) at −50 °C and 0.5 mbar for two days. The obtained powder was stored in the same vial at room temperature.

### 2.9. Antimicrobial Testing

#### 2.9.1. Preparation of the Inoculum

The antimicrobial properties of the TLE were tested against Gram-positive bacteria (*Staphylococcus aureus*; ATCC 10786), Gram-negative bacteria (*Escherichia coli*; ATCC 8739, and *Pseudomonas aeruginosa*; ATCC 27853), as well as one fungus (*Candida albicans*; ATCC 10231). Strains used in this study were sourced from the American Type Culture Collection (ATCC, Manassas, VA, USA). Confirmation of bacterial and fungal growth was identified based on morphological traits like colony appearance, color, and hemolysis on agar, along with Gram staining. The bacteria were sub-cultured onto MHA at 37 °C overnight, while the fungal inoculum was prepared from the 48 h culture of fungal isolates on SDA. Fresh microbial suspensions were prepared at 1 × 10^8^ colony forming units (CFU)/mL for the bacterial strains and 1 × 10^6^ CFU/mL for *C. albicans* [16,17].

#### 2.9.2. Antimicrobial Susceptibility Testing

The antimicrobial potential was assessed through the agar diffusion method according to Clinical & Laboratory Standards Institute (CLSI) guidelines after slight modifications [18,19]. Bacterial strains were swabbed onto MHA using sterile cotton swabs, while *C. albicans* inoculum was swabbed onto MHA supplemented with 2% glucose. The agar was then allowed to absorb the excess surface moisture for five minutes. Six wells, 6 mm in diameter, were cut out of the agar using a sterile cork borer. Freeze-dried TLE powder was dissolved in 10% DMSO/PBS at 40 mg/mL, followed by 2-fold serial dilution to prepare 20, 10, 5, 2.5, and 1.25 mg/mL solutions using PBS. Fifty microliters of each TLE concentration were placed into the corresponding wells. A Gentamicin (10 µg) disk was used as positive control except for *C. albicans* where a fluconazole (25 µg) disc was applied. Then, plates were kept at room temperature for one hour to enable diffusion of the extract then incubated at 37 °C for 24 h. DMSO at concentrations of 10%, 5%, 2.5%, and 1.25% in PBS, was used as the negative control. The antibacterial and antifungal activities were assessed by measuring inhibition zone surrounding the wells (including wells’ diameter) using a digital caliper. The test was performed in triplicate and outcomes were expressed as means ± SD.

### 2.10. Evaluation of the Anticancer Activity of TLE

The potential anticancer activity of the extract was evaluated against MCF-7 cells as a model breast cancer cell line. Cells were obtained from ATCC (Manassas, VA, USA). MCF-7 cells (HTB-22) were grown in Minimum Essential Medium (MEM; EuroClone, Pero, Italy) containing 10% heat-inactivated fetal bovine serum (FBS; EuroClone) and 1% penicillin–streptomycin (100 U/mL–100 mg/mL; EuroClone) at 37 °C in a humidified 5% CO_2_ incubator. To perform cytotoxicity experiments, cells were detached using trypsin-EDTA (EuroClone) and seeded in 96-well plates at 10,000 cells/well. Cells were allowed to be attached overnight, and then were treated with TLE at 2000, 1000, 500, 100, 10, and 1 µg/mL (diluted in the culture medium from a stock solution in PBS) for 72 h (5 replicates per concentration) [20]. The negative control group consisted of cells treated with the culture medium only [21]. Following the incubation period, the media was discarded from all the wells, followed by adding fresh medium containing 0.1 mg/mL resazurin (Santa Cruz Biotechnology, Dallas, TX, USA). After incubation for 2 h, the fluorescence intensity of the plate was measured using a Synergy HTX multimode reader (Biotek, Winooski, VT, USA) at 540 nm excitation and 620 nm emission wavelengths. Cell viability was expressed according to Equation (1) [22]:

(1) $$\text{Cell}\;\text{viability}\;\%\;=\frac{\text{Fluorescence}\;\text{of}\;\text{sample}\;\text{wells}}{\text{Fluorescence}\;\text{of}\;\text{negative}\;\text{control}\;\text{wells}}\times 100$$

The half-maximal inhibitory concentration (IC_50_) was then obtained by nonlinear regression analysis of cell viability % versus log concentration performed in GraphPad Prism version 9.5.1.

### 2.11. Evaluation of the Anti-Inflammatory Activity of TLE

The anti-inflammatory activity of TLE was evaluated by assessing its ability to scavenge intracellular reactive oxygen species (ROS) in stimulated RAW 264.7 macrophages using the 2′,7′-dichlorodihydrofluorescein diacetate (DCFDA; Abcam, Cambridge, UK) probe [23]. RAW 264.7 (TIB-71) macrophages were obtained from ATCC (Manassas, VA, USA) and grown in Dulbeccos’ Modified Eagle’s Medium (DMEM) containing 10% heat-inactivated FBS (EuroClone) and 1% penicillin–streptomycin (100 U/mL–100 mg/mL; EuroClone) at 37 °C in a humidified 5% CO_2_ incubator [24]. A cell viability experiment was performed first to select a nontoxic concentration range for the anti-inflammatory activity experiment. Cells were detached using a cell scraper and seeded in 96-well plates at 10,000 cells/well overnight. Cells were treated with TLE at 2000, 1000, 100, 10, and 1 µg/mL diluted in the culture medium from a stock solution in PBS for 24 h (5 replicates per concentration) [25]. The negative control group was composed of cells treated with the culture medium only. At the end of the incubation period, the media was removed from all wells, and cell viability was assessed as described above for MCF-7 cells.

For the anti-inflammatory activity experiment, the macrophages were seeded in 96-well plates at 10,000 cells/well overnight. The next day, cells were treated with 100, 10, and 1 µg/mL TLE diluted in the culture medium for 24 h (5 replicates per concentration). After 24 h, the DCFDA reagent was prepared at 40 µM in DMEM and mixed with an equal volume of 500 µM hydrogen peroxide (H_2_O_2_) in DMEM to induce intracellular ROS production. Then, 100 µL of this mixture (20 µM DCFDA/250 µM H_2_O_2_) was added to each well treated with TLE. The positive control group consisted of cells treated with DCFDA/H_2_O_2_ without prior treatment with TLE, and the negative control group consisted of cells treated with DCFDA only without prior treatment with TLE [26]. The plate was incubated for 45 min, followed by removing the media and adding 100 µL PBS to each well. The fluorescence of the plate was measured using a Synergy HTX multimode reader (Biotek, Winooski, VT, USA) at 485 nm excitation and 528 nm emission wavelengths [27]. The ability of TLE to scavenge intracellular ROS was expressed according to Equation (2):

(2) $$\text{ROS}\;\text{scavenging}\;\text{activity}=\frac{\text{Fluorescence}\;\text{of}\;\text{sample}\;\text{wells}}{\text{Fluorescence}\;\text{of}\;\text{negative}\;\text{control}\;\text{wells}}$$

where the fluorescence intensity is related to the cleavage of non-fluorescent DCFDA into a green fluorophore (DCF) in the presence of intracellular ROS, and a reduction in green fluorescence indicates ROS scavenging activity.

### 2.12. Analysis of TLE Constituents by Liquid Chromatography-Mass Spectroscopy

The identification of the bioactive compounds present in the TLE was achieved by liquid chromatography–mass spectroscopy (LC-MS) using a library of plant polyphenol standards. Standard stock solutions were obtained by dissolving the required amount of each substance in DMSO (analytical grade), followed by dilution with acetonitrile (LC/MS grade). The TLE sample was dissolved in DMSO (100 µL) and methanol (1.5 mL; LC/MS grade) and centrifuged at 4000× *g* for 2 min prior to analysis.

Analysis was performed using a Bruker Daltonik Impact II ESI-Q-TOF System equipped with a Bruker Daltonik Elute UPLC system (Bremen, Germany). The instrument utilized an Ion Source Apollo II ion Funnel electrospray source. The capillary voltage was 2500 V, the nebulizer gas was 2.0 bar, the dry gas (nitrogen) flow was 8 L/min and the dry temperature was 200 °C. The mass accuracy was ˂1 ppm, the mass resolution was 50,000 FSR (Full Sensitivity Resolution), and the TOF repetition rate was up to 20 kHz. Chromatographic separation was performed using a Bruker solo 2.0 C18 UHPLC column (100 mm × 2.1 mm × 2.0 μm) at a flow rate of 0.51 mL/min and a column temperature of 40 °C. Solvent A consisted of water supplemented with formic acid (0.1%), and solvent B was methanol. Injection volume was 3 µL. The standards were used to identify the constituents of the analyzed extract determined by the comparison of the m/z ratio and retention time after chromatographic separation.

### 2.13. Statistical Analysis

Experiments are conducted in triplicate with some repeated five times as mentioned in the results to ensure reproducibility and validity of the results acquired. Data is expressed as mean values ± standard deviations (SDs). Cell viability and anti-inflammatory activity results were analyzed in GraphPad Prism version 9.5.1, and the means were compared based on one-way ANOVA followed by Tukey’s post hoc test at *p* < 0.05. For RSM optimization, statistical analyses were performed using STATGRAPHICS Centurion XVI.I (Statgraphics 18, The Plains, VA, USA).

## 3. Results and Discussion

### 3.1. Determination of Solid-to-Liquid Extraction Ratio

In order to determine the optimal solid-to-liquid ratio for extraction, various ratios were tested and compared based on the TPC results. The optimization of the solid-to-liquid ratio is a critical step in maximizing the extraction of phenolic compounds, as an adequate solvent volume helps ensure a sufficient concentration gradient that promotes the diffusion of phenolics from the plant matrix into the solvent. Distilled water was used in this study as the extracting solvent with the solid-to-liquid ratios ranging from 1 g in 10 mL to 1 g in 70 mL, with all experiments adopting 20 min as the extraction time. The runs and their TPC results are presented in Table S1 in the supplementary material. The results showed an increase in TPC as the liquid volume increased, reaching a peak at a ratio of 1:50 (1 g in 50 mL), where the TPC measurement was 23.86 ± 0.10 mg GAE/g DM. Beyond this point, specifically at 1:60 and 1:70, the TPC values remained stable with a slight decrease at 1:60, indicating no further improvement in extraction efficiency beyond the mentioned point.

A smaller solid-to-liquid ratio generally results in better extraction efficiency, improving the diffusion of the compounds into the liquid solvent and accelerating mass transfer [28]. Similar observations are recurrent in the literature. In a study testing the extraction ratio (solid to liquid) for extracting polyphenols from the stem bark of *Funtumia elastica* (Funtum), it was found that raising the ratio from 1:10 to 1:30 significantly increased TPC yield, with a decline observed beyond this ratio at 1:40 and 1:50 [28]. Thus, while increasing the solvent volume can initially enhance the recovery of bioactive compounds, further addition becomes ineffective once the polyphenol content in the plant material is exhausted, resulting in no significant improvement in yield [29]. Therefore, the optimal solid-to-liquid ratio of 1:50 was used for all subsequent extraction processes in the current study.

### 3.2. Effect of Time and Temperature on TPC Yield and DPPH

To optimize polyphenol extraction, RSM was applied to identify the optimum conditions for WBE, targeting the highest possible TPC and antioxidant activity. In this experimental design, the solid-to-liquid ratio was maintained at 1:50 (g/mL), while the extraction temperature and time were varied to develop the predictive model. The responses of TPC and antiradical activity were analyzed according to the Pareto charts and 3D mesh. Table 1 summarizes the performed runs on the TLE obtained by WBE and presents their corresponding TPC and DPPH results.

**Figure 1:**
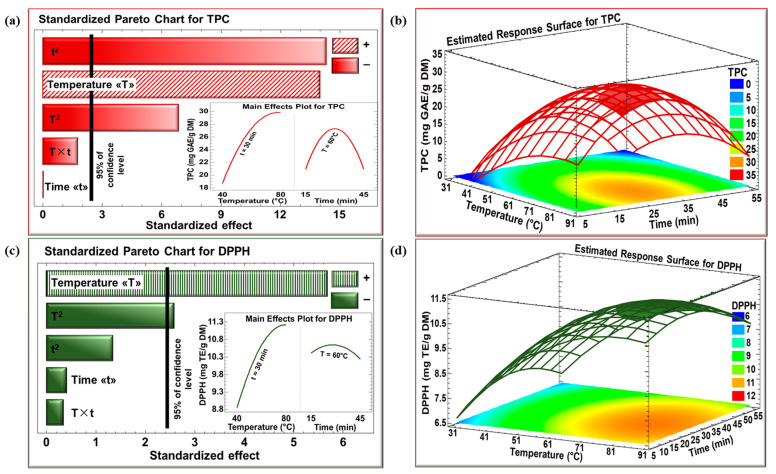
Standardized Pareto Charts with inserts for the effect of temperature and time on (**a**) TPC and (**c**) DPPH of TLE and estimated response surface for (**b**) TPC and (**d**) DPPH of TLE. (+) indicates a positive effect, and (−) indicates a negative effect.

The effects of temperature and time on the TPC and DPPH scavenging activity of TLEs were analyzed using Pareto charts and response surface plots, as illustrated in Figure 1. In the Pareto charts, any horizontal bar extending beyond the threshold line indicates a statistically significant effect at a confidence level greater than 95%. Figure 1a shows that temperature had a significant positive linear effect on the TPC of TLE, with values increasing from almost 8.5 mg GAE/g DM to 29 mg GAE/g DM. However, a significant negative quadratic effect was recorded beyond 80 °C. Regarding extraction time, a significant negative quadratic effect was also observed. TPC increased from 15 min, peaked at approximately 30 min, and then decreased to 21 mg GAE/g DM at 45 min. Hence, prolonged extraction time beyond 30 min negatively influenced the TPC yield. Figure 1b confirms the trends observed in Figure 1a regarding the effects of temperature and time, and further illustrates the optimal conditions for maximizing TPC, which reached nearly 30 mg GAE/g DM. The red region in the plot represents the most effective combinations of temperature and time for achieving the highest TPC levels.

As for the Pareto chart of DPPH scavenging activity (Figure 1c), temperature had a significant positive linear effect, with DPPH values increasing from 8.8 to 11.2 mg TE/g DM between 40 and approximately 80 °C. Beyond this point, a negative quadratic effect was observed (inset of Figure 1a). Time had no significant effect on DPPH of TLE. These results suggest that temperature plays a significant role in optimizing antioxidant yield, while prolonging the extraction process does not necessarily enhance DPPH scavenging activity. Figure 1d confirms the trend observed in Figure 1b and highlights the ideal conditions for DPPH scavenging activity, where the green region indicates combinations that yielded the most DPPH scavenging activity (about 11 mg TE/g DM).

The increase in extraction temperature enhances mass transfer by improving solute solubility, reducing solvent viscosity, and increasing diffusion coefficients, all of which contribute to higher extraction efficiency [30]. This effect was observed in this study, where temperature exhibited a positive linear impact on TPC levels and antioxidant activity. However, beyond 80 °C, temperature showed a negative quadratic effect on both the TPC and DPPH values of TLE. This finding is consistent with previous studies using conventional water bath extractions, where the highest yields of phenolic compounds were typically achieved at temperatures between 60 and 80 °C [31]. The observed decline may be a result of heat-induced degradation of phenolic compounds after prolonged exposure at higher temperatures [32].

RSM allowed the optimization of the response parameters (TPC and DPPH) and the development of a second-degree mathematical model for predicting their values through statistical analysis. The regression equations fitted to the experimental data are presented in Table 2, with variables expressed in their original units. Two sets of equations are provided: the first includes all effects: linear, interaction, and quadratic, while the second represents only the variables that had a statistically significant impact. For example, time and the interaction between time and temperature did not have a significant effect on TPC extraction and were therefore removed from the simplified equation. Similar approaches have been reported in literature, where multiple-response optimizing using temperature-time combinations was successfully applied for maximizing phenolic recovery from grape byproducts [33].

**Figure 2:**
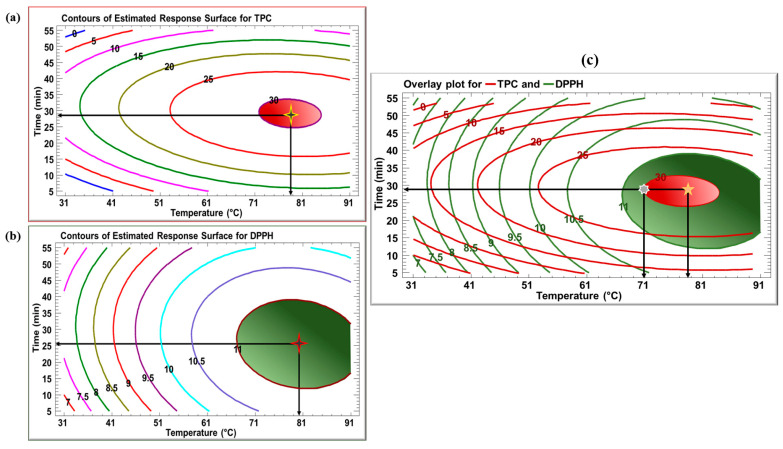
Contours of estimated response surface for (**a**) TPC and (**b**) DPPH of TLE as a function of time and temperature; (**c**) Overlay plot for TPC and DPPH of TLE as a function of time and temperature.

Figure 2 exhibits the contours of estimated response surfaces of TPC and DPPH as a function of time and temperature. The objective of this analysis is to determine the optimal conditions for maximizing TPC and DPPH scavenging activity during the extraction process. For TPC, the contours represent various levels of TPC ranging from 0 to 30 mg GAE/g DM with the highest concentration represented in the red zone. The figure shows that the optimum TPC yield of around 30 mg GAE/g DM is achieved at 78.5 °C and 29 min. This point signifies the combination of extraction conditions under which the maximum TPC yield was achieved. For DPPH, the plot displays that the optimum level of 11 mg TE/g DM is achieved at 80 °C and 25.5 min. This is the point where the most efficient combination of time and temperature achieved the highest antioxidant potential. To confirm the validity of the prediction model, extraction was carried out under the predicted optimum conditions, and TPC and DPPH scavenging activity were determined experimentally. The TLE yielded a TPC of 27.7 mg GAE/g DM, and a DPPH scavenging activity of 10.22 mg TE/g DM, which were very similar to the values predicted by the model.

The optimal extraction conditions for the TLE are summarized in Table 3, where the model yielded good satisfactory adequacy as reflected by high R^2^ values of 87% and 95%. Figure 2c shows the overlay plot for the TPC (red) and DPPH (green) of TLE. The overlapping zone for TPC and DPPH (between 71 and 81 °C at 29 min) includes the optimal conditions of 78.5 °C and 28.9 min, indicating a favorable compromise for maximizing both phenolic yield and antioxidant activity. Although these are the achieved results according to the overlay plot, a temperature of 71 °C for the same duration of 29 min can also be used, as it falls within the overlapping region of the optimal TPC and DPPH. While the values may be slightly lower, this adjustment allows for reduced temperature usage, contributing to energy conservation. DPPH scavenging activity analysis using ascorbic acid was carried out to further confirm the Trolox trials and revealed similar results, further confirming the antioxidant activity. Results are presented in Table 1.

The optimum extracted concentration of 27.7 mg GAE/g DM, using only distilled water as the extracting solvent, is comparable to other studies using leafy materials to extract phenolic compounds. In a study on olive leaves, phenolic compounds were extracted using the water bath method with ethanol as the solvent, yielding a TPC of 11.67 mg GAE/g DW, significantly lower than that obtained from the tomato leaves using distilled water es the extracting solvent [34]. Similarly, *Carica* papaya leaves were evaluated for TPC using distilled water as the solvent and water bath extraction, yielding 23.06 ± 1.06 mg GAE/g DW, slightly lower than the value obtained by tomato leaves in the current study [35].This suggests that TLE not only serves as a potent source of polyphenol but also supports the use of low-cost, environmentally friendly extraction approaches that minimize the need for complex equipment or hazardous solvents.

### 3.3. Antimicrobial Activity of TLE

No antimicrobial activity was observed against *P. aeruginosa* or *S. aureus* (zone of inhibition of 0), while concentration-dependent inhibitory activity was observed in *E. coli* and *C. albicans* using the lyophilized extract (Table 4). The negative control (DMSO/PBS only with no TLE) did not show antibacterial activity as evident from the absence of clear zones of growth inhibition, confirming that the observed activity was attributed to the extract. As shown in Table 4, the zone of inhibition against *E. coli* ranged from 17.6 ± 1.5 mm at 40 mg/mL to 8.6 ± 0.5 mm at 2.5 mg/mL. At 1.25 mg/mL, no inhibition zone was observed. As for *C. albicans*, inhibition zones ranged from 21.4 ± 0.4 mm to 9.6 ± 0.1 mm at 40 mg/mL and 5 mg/mL, respectively. However, TLE concentrations of 2.5 and 1.25 mg/mL did not exhibit activity. Figures S1 and S2 show the dose-dependent zones of inhibition for *E. coli* and *C. albicans*, respectively.

These findings add to what is found in the literature regarding the antimicrobial activity of tomato leaves. For example, a study evaluated the antimicrobial activity of the acetonic extract of tomato leaves and found that it exhibited inhibitory effects against fungi and oomycetes pathogenic to tomato plants including *Colletotrichum coccodes*, *Fusarium oxysporum* f. sp. *Lycopersici*, and *Phytophthora capsici.* The extract also demonstrated activity against pathogens infecting strawberry plants (*Glomerella cingulata*, *Phytophthora cactorum*, and *Rhizoctonia solani*) that was the most susceptible to the extract [9]. Another study evaluated the antibacterial activity of various parts of the tomato plant including the leaves against *E. coli* O157:H7, *S. typhimurium*, *S. aureus*, and *L. ivanovii* [8]. Two cultivars of tomato plant were tested, Pitenza and Floradade, and the leaves of both plants exhibited antibacterial effects against all tested strains with inhibition zones ranging between 9.1 mm to 12.9 mm. Furthermore, the antifungal activity reported for TLE in the current study is notable. A study that investigated the antifungal activity of the ethanolic leaf extract showed no inhibitory effect against *C. albicans*, in contrast to our findings [36]. This could be attributed to variations in plant source (Leaves of Chonto tomato versus Plum tomato in this report), in addition to disparities in environmental factors, cultivation and growing conditions, as well as strain sensitivity. These results, along with the current findings, suggest that tomato leaves contain bioactive compounds capable of disrupting microbial growth, particularly in fungal organisms such as *E. coli* and bacteria. Moreover, comparing TLE’s efficacy to standard antibiotics such as gentamicin and fluconazole can help highlight its potential as a complementary or alternative antimicrobial agent, especially in an era where natural products are being revisited for their therapeutic value against resistant strains. This antimicrobial activity can be attributed to the bioactive compounds present in the extract. For example, rutin and quercetin, two of the molecules present in the extract, are known to have antimicrobial activity against various Gram-positive and negative bacteria including *E. coli* and *S. aureus* as well as other bacteria and fungi [37]. Indeed, while the mentioned compounds represent part of the extract’s constituents, the effect would not be attributed solely to them, rather it is the effect of the combined actions of the multiple constituents rather than a single compound.

### 3.4. Anticancer Activity of TLE Against MCF-7 Cells

**Figure 3:**
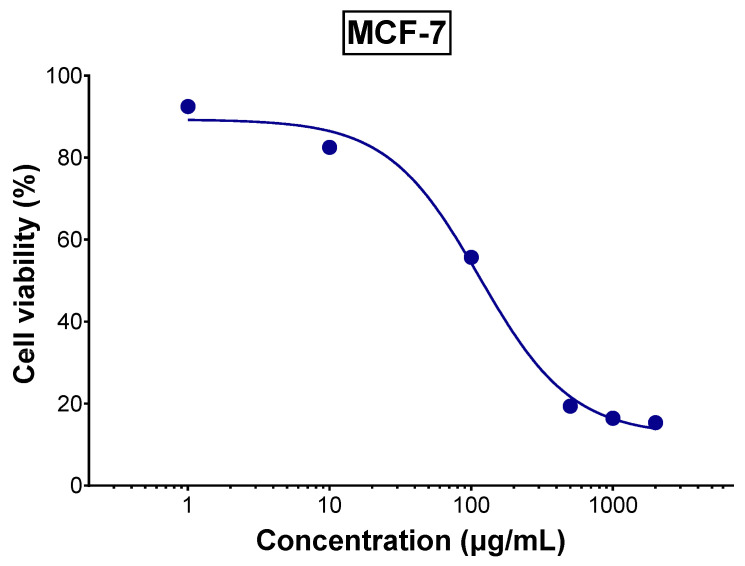
Viability of MCF-7 cells treated with various concentrations of TLE for 72 h (*n* = 5). (Error bars too small).

The aqueous extract of tomato leaves demonstrated promising anticancer activity against MCF-7 breast cancer cells with an IC_50_ of 114.5 µg/mL as shown in Figure 3, indicating moderate cytotoxic potential. This activity is likely attributed to the polyphenol and alkaloid constituents in the extract. Previous research has shown that tomatidine and tomatidine-rich tomato leaf extract can significantly inhibit tumor development in vivo and reduce the proliferation of human gastric cancer-derived 85As2 cells by modulating type I interferon-stimulated gene expression [10]. Additionally, rutin, identified as the most abundant polyphenol present in TLE as identified by LC-MS, has been shown to regulate multiple key cell signaling pathways associated with onset and progression of cancer, such as PI3K/Akt, MAPK, and NF-κB pathways, suggesting its potential role in enhancing anticancer activity [38]. Also, α-tomatine, another constituent of the extract, was shown to have a cytotoxic effect on MCF-7 breast cancer cells primarily through non-apoptotic mechanisms, including ATP depletion, rather than via DNA damage or caspase-mediated apoptosis [39].

These findings suggest that the anticancer effects of tomato leaf extract may result from the synergistic interaction between its bioactive polyphenolic compounds and steroidal alkaloids. Both classes of compounds have individually shown cytotoxic effects against cancer cells in previous studies. Such a multifaceted activity provides a strong rationale for further investigation into TLE’s potential as a natural therapeutic agent against cancer. Notably, the extract exhibited selective cytotoxicity at concentrations that preserved normal macrophage viability (as seen in the anti-inflammatory assay) at equivalent concentrations, suggesting a potentially favorable safety profile.

### 3.5. Anti-Inflammatory Activity of TLE in RAW 264.7 Macrophages

**Figure 4:**
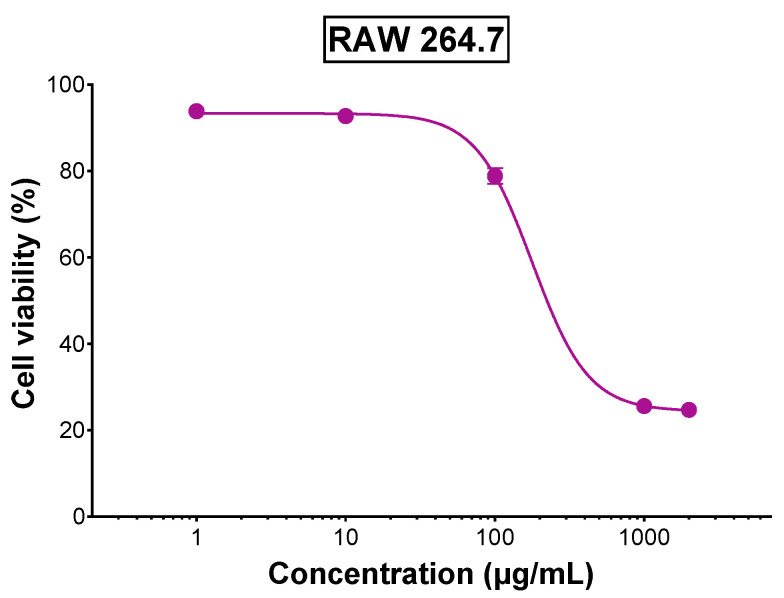
Viability of RAW 264.7 macrophages treated with various concentrations of TLE for 24 h (*n* = 5). (Error bars are too small).

**Figure 5:**
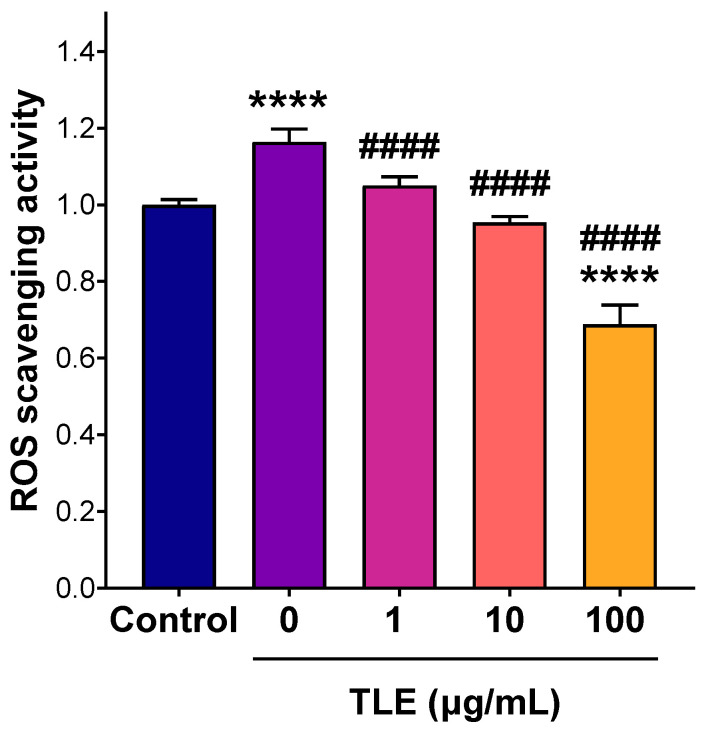
Ability of TLE to scavenge intracellular ROS measured by a DCFDA assay in RAW 264.7 macrophages. Cells were treated with different concentrations of TLE for 24 h and then stimulated with H_2_O_2_. Intracellular ROS levels were assessed by measuring dichlorofluorescein (DCF) fluorescence relative to the negative control and are expressed as the mean ± standard deviation (*n* = 5). **** *p* < 0.0001 compared to the untreated/unstimulated negative control group; ^####^ *p* < 0.0001 compared to the untreated/stimulated (0 µg/mL TLE) positive control group. Statistical analysis was based on one-way ANOVA followed by Tukey’s post hoc test performed in GraphPad Prism 9.5.1.

Cytotoxicity of the extract on RAW 264.7 macrophages was tested to select a nontoxic concentration for the anti-inflammatory assay (Figure 4). The dose–response curve revealed that cell viability remained over 80% for concentrations up to 100 µg/mL, indicating minimal cytotoxicity at these doses. However, cell viability declined beyond 100 µg/mL suggesting potential cytotoxicity effects at higher concentrations. Thus, TLE concentrations of 1, 10, and 100 µg/mL were selected to evaluate the extract’s anti-inflammatory potential. Anti-inflammatory activity was examined by evaluating the extract’s capacity to protect the cells from ROS. As shown in Figure 5, untreated and stimulated cells (positive control) demonstrated a marked elevation in intracellular ROS relative to the unstimulated negative control (*p* < 0.0001). However, treatment with 1 and 10 µg/mL of TLE showed a significant reduction in intracellular ROS compared to the positive control group (*p* < 0.0001), where the levels were close to the negative control. Moreover, the highest concentration (100 µg/mL) exhibited significantly lower intracellular ROS levels compared to the negative control, indicating the ability of TLE to relieve basal ROS levels. Given that elevated levels of ROS are contributing factors to multiple disorders including inflammation, the results further supported the anti-inflammatory activity of TLE. These findings highlight the dual function of TLE; it is non-toxic at relevant concentrations and effective in mitigating oxidative stress as well as possessing an anticancer activity. The anti-inflammatory properties of TLE align with findings from studies on other plant extracts such as *Lasia spinosa* leaf extract that exhibited anti-inflammatory activity in lipopolysaccharide-induced RAW 264.7 macrophages by significantly reducing ROS and pro-inflammatory mediator, an effect attributed to its high polyphenolic content and antioxidant potential [40]. The extract also modulated key signaling pathways, including NF-κB and PI3K/Akt, highlighting the therapeutic potential of plant-derived polyphenols in reducing oxidative stress and inflammation. Additionally, α-tomatine, a major constituent present in tomato leaf extract, has been reported to exhibit anti-inflammatory activity by significantly suppressing pro-inflammatory cytokine production in lipopolysaccharide-induced macrophages [41]. This effect was associated with the inhibition of NF-κB-p65 nuclear translocation and ERK1/2 phosphorylation, alongside enhanced Akt phosphorylation, suggesting that α-tomatine may contribute to the anti-inflammatory properties of tomato leaf extract through modulation of key inflammatory signaling pathways.

### 3.6. LC-MS Analysis of TLE

The LC-MS analysis of TLE indicated the occurrence of various bioactive compounds in different proportions as presented in Table 5. Among the identified compounds, rutin was present in the highest amount making up about 45.28% of the total detected compounds, followed by 4-hydroxycoumarin (13.62%), α-tomatine (12.40%), and dehydrotomatine (9%). Other identified compounds included quercetin (6.74%), chlorogenic acid (5.66%), and 4-methylumbelliferone (4.78%), all of which are associated with notable antioxidant and pharmacological properties. Some compounds were detected in minor levels including aesculetin (1.90%), capsaicin (0.2%), pinolenic acid (0.15%), (Z)-3-hydroxyoctadec-7-enoic acid (0.14%), and artocaprin (0.13%). Figures S3–S5 show the chromatograms of the identified compounds. As shown in Figure S3, some unidentified peaks with notable intensities appeared between 19 and 20 min, which could not be properly assigned due to the limitations/availability of the standards. However, as mentioned previously, the highest among the detected compounds was rutin. The results are in concordance with previous findings where considerable amounts of rutin, gallic acid, and chlorogenic acid were detected in tomato leaves [8]. Rutin is known for its anti-inflammatory, anticancer, antioxidant, and antidiabetic properties [42]. These properties are attributed to their ability to scavenge free radicals [43], modulate inflammatory pathways [44], and regulate key enzymes involved in metabolic disorders [45]. Its presence in TLE not only enhances the therapeutic value of the extract but also positions TLE as a promising candidate of functional foods, nutraceuticals, and natural health products. Additionally, the synergistic effects of rutin with other phytochemicals present in the extract may further enhance its bioactivity, supporting its use in pharmaceutical formulations as well as for other applications.

## 4. Conclusions

This study demonstrates the potential of tomato leaves, an often discarded agricultural by-product, as a sustainable source of bioactive compounds with significant biological activities. Through optimized water bath extraction, the optimal yield of phenolic compounds and antioxidant activity were achieved with best conditions being at 71 °C for 29 min and a 1:50 solid-to-liquid ratio using distilled water as the extracting solvent. The lyophilized tomato leaf extract exhibited antimicrobial activity against *E. coli* and *C. albicans*, anticancer effects against MCF-7 breast cancer cells, and strong anti-inflammatory potential through effective scavenging of intracellular ROS in RAW 264.7 macrophages. LC-MS analysis confirmed the presence of bioactive compounds such as rutin, 4-hydroxycoumarin, α-tomatine, and dehydrotomatine, which may act synergistically to enhance these biological effects. Unlike previous studies that relied on organic solvents or assessed limited biological property, our work provides the first integrated approach that combines green extraction, statistical optimization, bioactivity evaluation, and LC–MS characterization, thereby setting a precedent for sustainable tomato leaf valorization. Taken together, these findings not only validate the valorization of tomato leaves as a functional ingredient for pharmaceutical or nutraceutical applications but also highlight the broader implications of agricultural waste utilization in promotion of circular economy.

## Figures and Tables

**Table 1:** Central composite design for time and temperature with the corresponding TPC and DPPH experimental responses of TLE.

	Run	Temperature (T) °C	Time (t) Min	TPC (mg GAE/g DM)	DPPH (mg TE/g DM)	DPPH AA (mg AAE/g DM)
Factorial Points	1 2 3 4	40 (−1) 80 (+1) 40 (−1) 80 (+1)	15 (−1) 15 (−1) 45 (+1) 45 (+1)	11.03 ± 0.15 23.58 ± 0.39 12.77 ± 0.18 21.39 ± 0.77	7.74 ± 0.05 11.00 ± 0.09 7.96 ± 0.16 10.81 ± 0.10	6.92 ± 0.04 10.09 ± 0.08 7.14 ± 0.15 9.88 ± 0.09
Star Points	5 6 7 8	32 (–α) 88 (+α) 60 (0) 60 (0)	30 (0) 30 (0) 8.8 (–α) 51.2 (+α)	13.57 ± 0.41 30.16 ± 0.39 15.04 ± 0.15 15.40 ± 0.29	8.57 ± 0.07 10.98 ± 0.10 10.61 ± 0.02 10.10 ± 0.11	7.72 ± 0.07 10.04 ± 0.10 9.68 ± 0.02 9.19 ± 0.11
Center Points	9 10 11 12	60 (0) 60 (0) 60 (0) 60 (0)	30 (0) 30 (0) 30 (0) 30 (0)	27.50 ± 0.35 26.62 ± 0.27 28.68 ± 0.79 26.40 ± 0.38	10.76 ± 0.04 10.30 ± 0.06 10.65 ± 0.11 10.86 ± 0.04	9.82 ± 0.03 9.39 ± 0.6 9.72 ± 0.11 9.92 ± 0.04

**Table 2:** Second-order regression equations of TPC and DPPH. (1) and (3) are the general equations and (2) and (4) are the equations with the significant parameters.

(1) TPC = −48.4 + 1.29 × T + 1.9 × t − 0.007 × T^2^ − 0.003 × T × t − 0.028 × t^2^ (2) TPC = −42.45 + 1.19 × T + 1.7 × t − 0.007 × T^2^ − 0.028 × t^2^
(3) DPPH = −0.082 + 0.25× T + 0.098 × t − 0.0015 × T^2^ − 0.0003 × T × t − 0.0014× t^2^ (4) DPPH = 2.11 + 0.22 × T − 0.0057 × t − 0.0013 × T^2^

**Table 3:** Optimum Extraction Conditions for TLE.

	Parameters	TPC	DPPH
Optimized operating conditions	Time (min)	29	29
	Temperature (°C)	78.5	78.5
Model-predicted optimal TPC and DPPH under optimized parameters	TPC optimal value (mg GAE/g DM)	30	-
	DPPH optimal value (mg TE/g DM)	-	11
Validation of model optimum under optimized conditions	TPC observed value (mg GAE/g DM)	27.7	-
	DPPH observed value (mg TE/mL)		10.22
	Model’s R-squared (%)	96	87

**Table 4:** Antimicrobial activity of TLE against *E. coli* and *C. albicans* presented as zones of inhibition (mm) after an agar well diffusion method (mean ± SD; *n* = 3).

Microbial Strain	TLE Concentration (mg/mL)	Zone of Inhibition (mm ± SD)
*E. coli*	40	17.6 ± 1.5
	20	15.2 ± 0.6
	10	14.3 ± 0.3
	5	10.7 ± 0.3
	2.5	8.6 ± 0.5
	1.25	0
	Gentamicin (10 µg)	22.4 ± 0.4
*C. albicans*	40	21.4 ± 0.4
	20	17.7 ± 1.7
	10	12.0 ± 2.0
	5	9.6 ± 0.1
	2.5	0
	1.25	0
	Fluconazole (25 µg)	30.4 ± 0.3

**Table 5:** Identified compounds in tomato leaf extract by LC-MS showing the retention times, m/z, measured molar masses, and percentages relative to the total identified compounds.

Compound	Retention Time (min)	m/z	Measured Molar Mass	Percentage (%) ^a^
Rutin	12.8	611.16	610.15	45.28
4-Hydroxycoumarin	4.47	163.039	162.03	13.62
α-tomatine	19.47	1034.54	1033.54	12.40
Dehydrotomatine	18.88	1032.52	1031.53	9
Quercetin	12.8	303.05	302.04	6.74
Chlorogenic acid	4.48	355.10	354.09	5.66
4-Methylumbelliferone	6.08	177.0	176.04	4.78
Aesculetin	6.36	179.03	178.02	1.90
Capsaicin	12.2	306.20	305.19	0.20
Pinolenic acid	27.9	281.24	280.23	0.15
(Z)-3-Hydroxyoctadec-7-enoic acid	26.85	299.25	298.24	0.14
Artocaprin	26.06	437.19	436.18	0.13

^a^ Calculated as the peak area of each compound relative to the total peak areas of all identified compounds × 100%.

## Data Availability

The original contributions presented in the study are included in the article, further inquiries can be directed to the corresponding author.

## Supplementary Materials

The following supporting information can be downloaded at https://www.mdpi.com/article/10.3390/foods14193383/s1, Figure S1: Representative agar plates showing zones of inhibition of TLE at concentrations of 40, 20, 10, and 5 mg/mL (left) and 2.5 and 1.25 mg/mL (right), along with gentamicin (right), against *E. coli*. Figure S2: Representative agar plates showing zones of inhibition of TLE at concentrations of 5, 2.5, and 1.25 mg/mL (left) and 40, 20, and 10 mg/mL (right), along with fluconazole (right), against *C. albicans*. Figure S3: LC-MS chromatogram of the tomato leaf extract acquired on a Bruker Impact II Q-TOF in the positive ion mode at a resolution of 50,000 FSR. Figure S4: LC-MS chromatograms of identified compounds (intensity vs. retention time) acquired on a Bruker Impact II Q-TOF in the positive ion mode at a resolution of 50,000 FSR. Figure S5: LC-MS chromatograms of identified compounds (intensity vs. m/z) acquired on a Bruker Impact II Q-TOF in the positive ion mode at a resolution of 50,000 FSR.Table S1: Influence of Solid-to-Liquid Ratio on Total Phenolic content (TPC) Extracted from Tomato Leaves. Table S2: Standards used for LC-MS analysis.
