# Supplementary material for: Valorization of Tomato Leaves: Optimization of Eco-Friendly Phenolic Extraction and Assessment of Biological Activities
Source: Foods. 2025 Sep 30;14(19):3383. doi: 10.3390/foods14193383 (PMC12523334; doi:10.3390/foods14193383)
Supplement: Supplementary file 1 [file foods-14-03383-s001.zip › foods-3840969-supplementary.pdf]

Supplementary Material

Table S1. Influence of Solid-to-Liquid Ratio on Total Phenolic content (TPC) Extracted from Tomato Leaves.

Run	Solid-to-liquid ratio (g/mL)	TPC (mg GAE/g DM)
**1**	1:10	10.39 ± 0.16
**2**	1:20	12.07 ± 0.11
**3**	1:30	15.02 ± 0.25
**4**	1:40	20.11 ± 0.26
**5**	1:50	23.86 ± 0.10
**6**	1:60	20.50 ± 0.34
**7**	1:70	23.37 ± 0.29

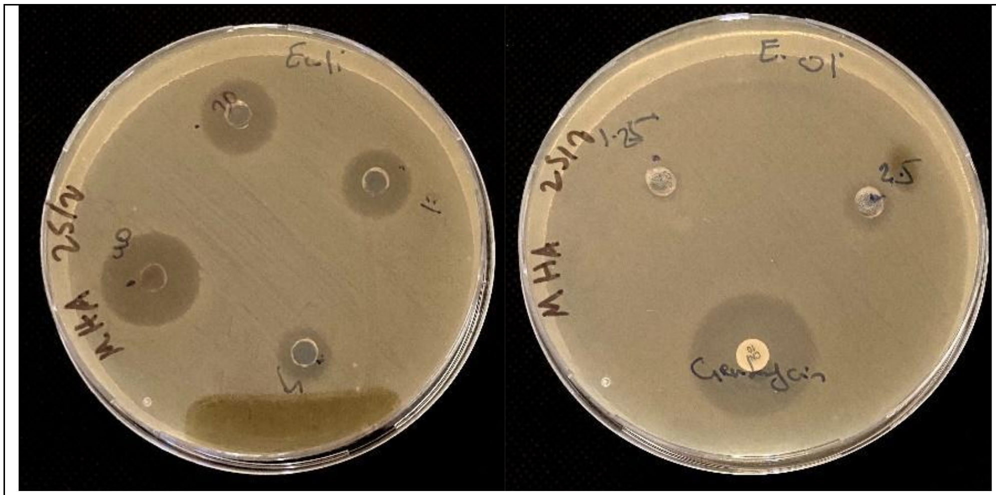

Figure S1. Representative agar plates showing zones of inhibition of TLE at concentrations of 40, 20, 10, and 5 mg/mL (left) and 2.5 and 1.25 mg/mL (right), along with gentamicin (right), against *E. coli*.

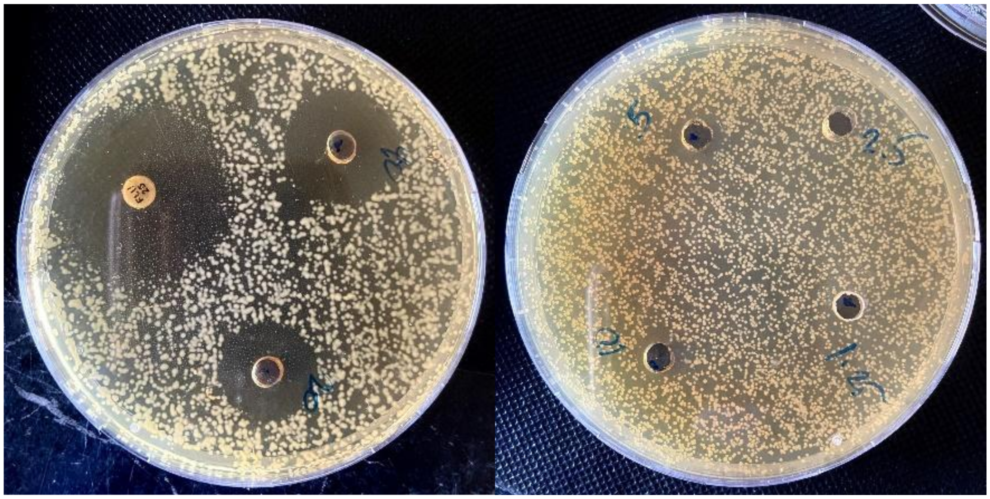

Figure S2. Representative agar plates showing zones of inhibition of TLE at concentrations of 5, 2.5, and 1.25 mg/mL (left) and 40, 20, and 10 mg/mL (right), along with fluconazole (right), against *C. albicans*.

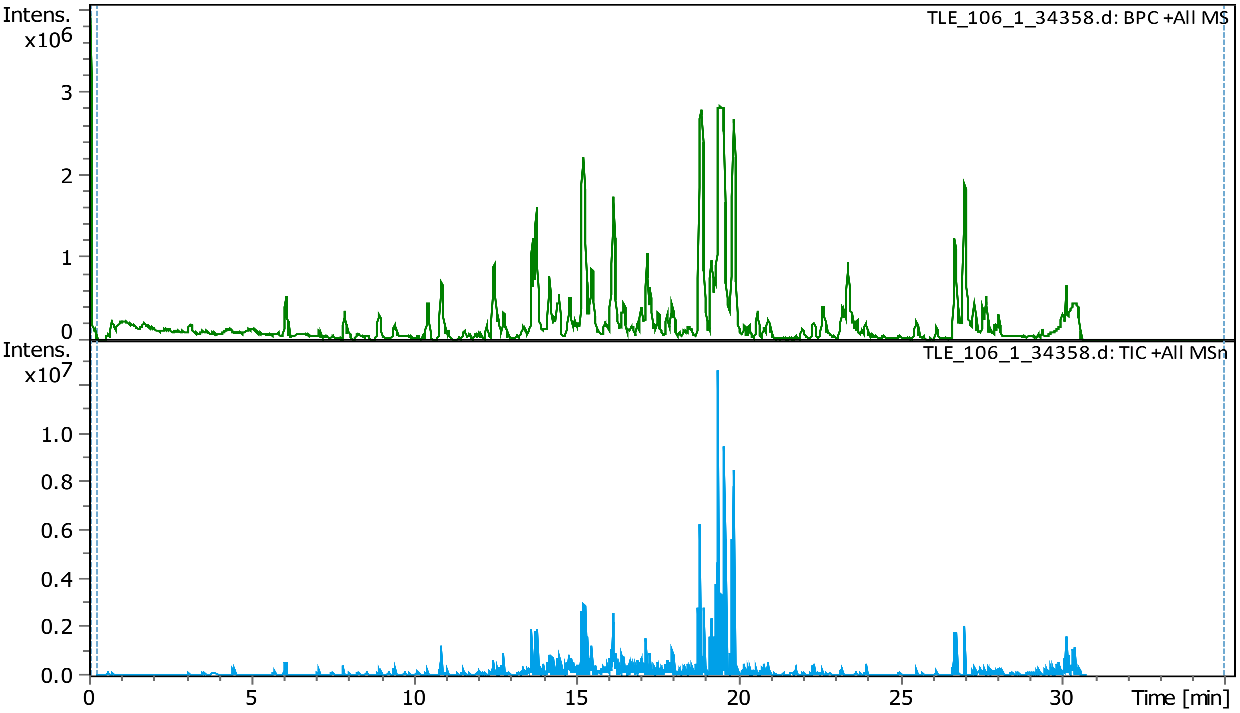

Figure S3. LC-MS chromatogram of compounds present in tomato leaf extract

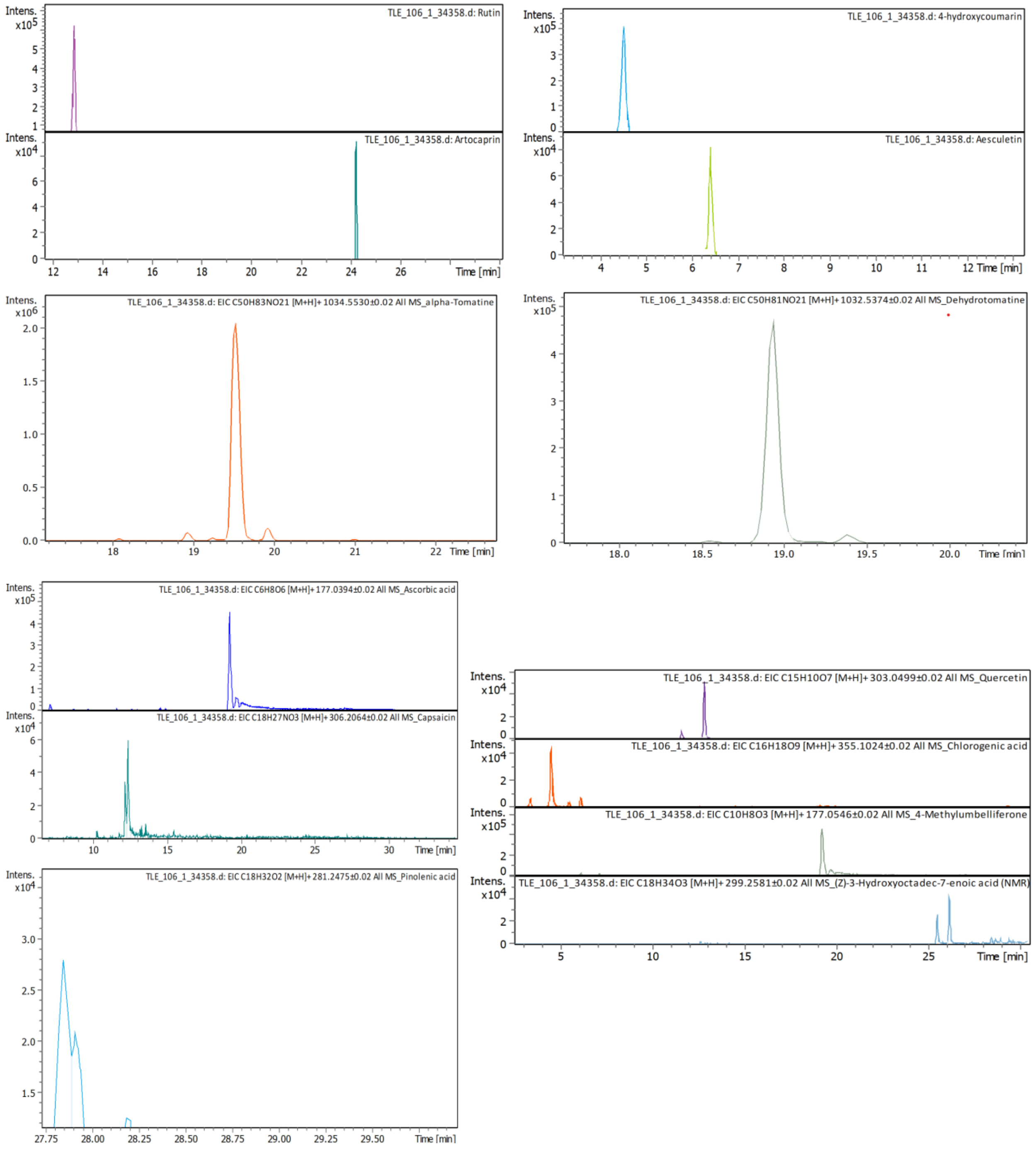

Figure S4. LC-MS chromatograms of identified compounds (intensity vs. retention time)

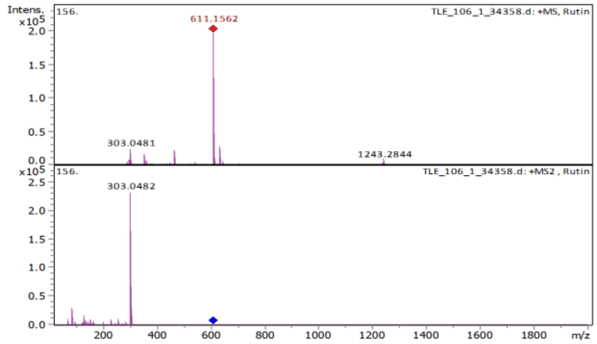
Intens.
x10^5
156.
TLE_106_1_34358.d: +MS, Rutin
611.1562
303.0481
1243.2844
156.
TLE_106_1_34358.d: +MS2 , Rutin
303.0482
m/z

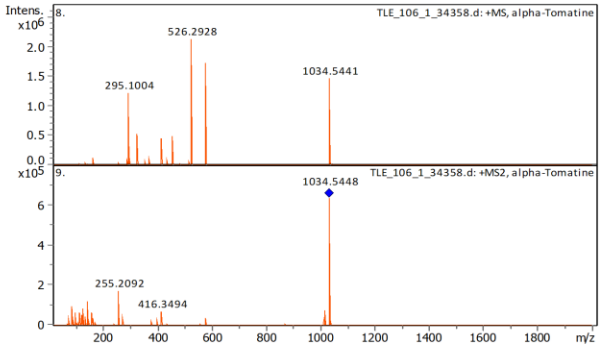
Intens.
x10^6
8.
TLE_106_1_34358.d: +MS, alpha-Tomatine
526.2928
295.1004
1034.5441
x10^5
9.
TLE_106_1_34358.d: +MS2, alpha-Tomatine
1034.5448
255.2092
416.3494
m/z

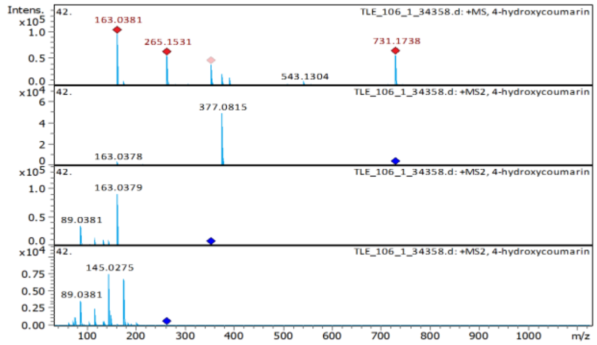
Intens.
x10^5
42.
TLE_106_1_34358.d: +MS, 4-hydroxycoumarin
163.0381
265.1531
731.1738
543.1304
x10^4
42.
TLE_106_1_34358.d: +MS2, 4-hydroxycoumarin
377.0815
163.0378
x10^5
42.
TLE_106_1_34358.d: +MS2, 4-hydroxycoumarin
163.0379
89.0381
x10^4
42.
TLE_106_1_34358.d: +MS2, 4-hydroxycoumarin
145.0275
89.0381
m/z

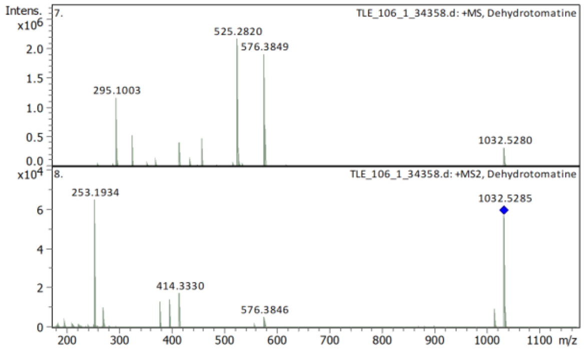
Intens.
x10^6
7.
TLE_106_1_34358.d: +MS, Dehydrotomatine
525.2820
576.3849
295.1003
1032.5280
x10^4
8.
TLE_106_1_34358.d: +MS2, Dehydrotomatine
253.1934
1032.5285
414.3330
576.3846
m/z

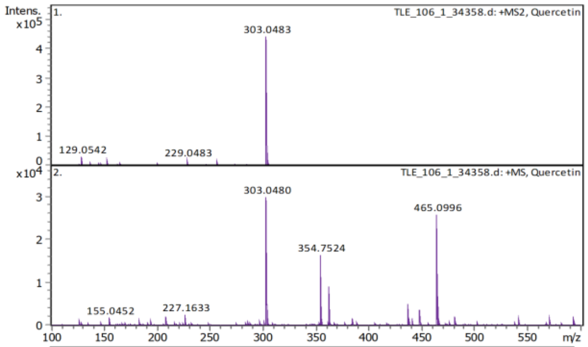
Intens.
x10^5
1.
TLE_106_1_34358.d: +MS2, Quercetin
303.0483
129.0542
229.0483
x10^4
2.
TLE_106_1_34358.d: +MS, Quercetin
303.0480
465.0996
354.7524
155.0452
227.1633
m/z

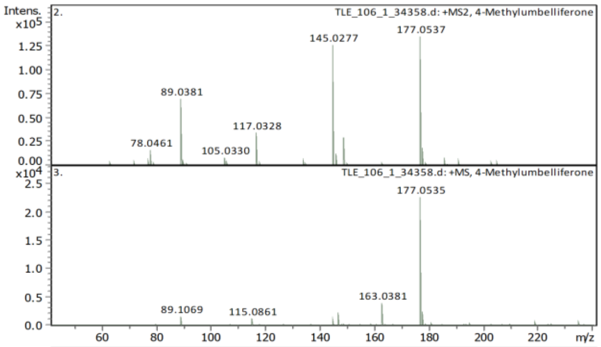
Intens.
x10^5
2.
TLE_106_1_34358.d: +MS2, 4-Methylumbelliferone
145.0277
177.0537
89.0381
117.0328
78.0461
105.0330
x10^4
3.
TLE_106_1_34358.d: +MS, 4-Methylumbelliferone
177.0535
163.0381
89.1069
115.0861
m/z

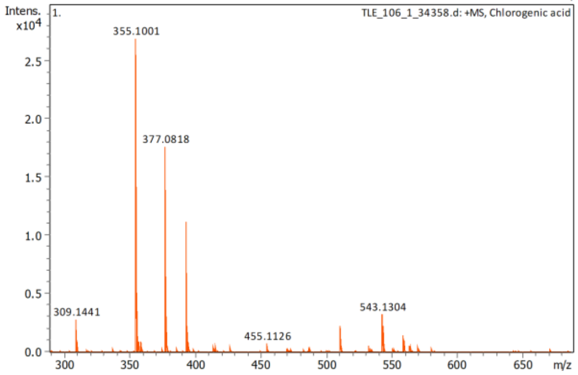
Intens.
x10^4
1.
TLE_106_1_34358.d: +MS, Chlorogenic acid
355.1001
377.0818
309.1441
455.1126
543.1304
m/z

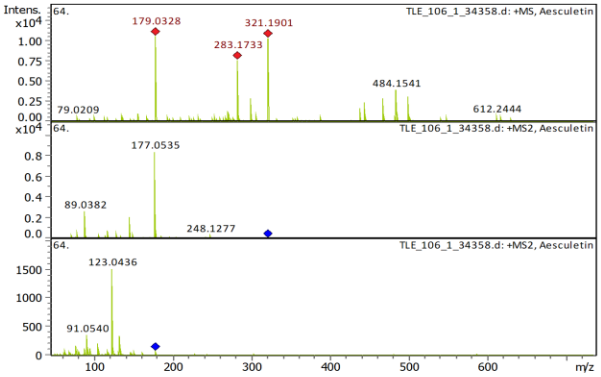
Intens.
x10^4
64.
TLE_106_1_34358.d: +MS, Aesculetin
179.0328
321.1901
283.1733
484.1541
79.0209
612.2444
x10^4
64.
TLE_106_1_34358.d: +MS2, Aesculetin
177.0535
89.0382
248.1277
64.
TLE_106_1_34358.d: +MS2, Aesculetin
123.0436
91.0540
m/z

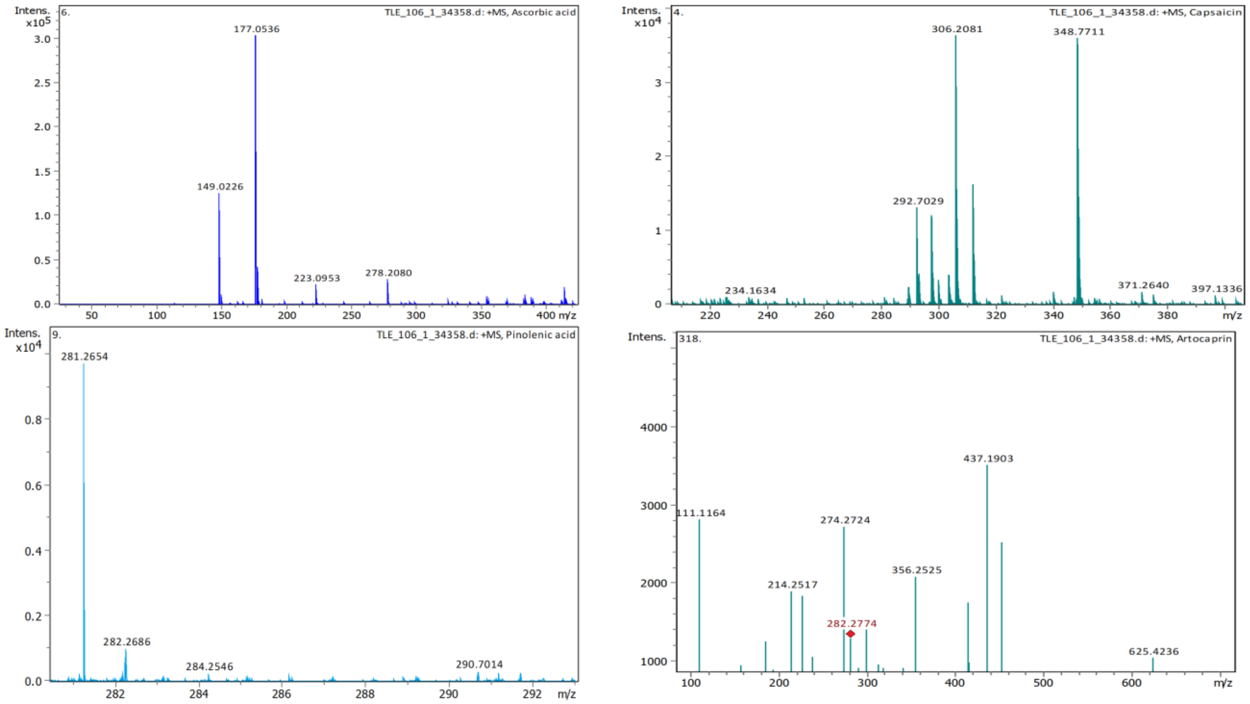

Figure S5. LC-MS chromatograms of identified compounds (intensity vs. m/z)

Table S2. Standards used for LC-MS analysis

No	Standard Name
1	Pivalic Acid
2	Catechol
3	Hydroquinone
4	Succinic acid
5	Benzoic acid
6	Cinnamic acid
7	Anisic acid
8	Vanillin
9	4-hydroxycoumarin
10	Umbelliferone
11	o-Coumaric acid
12	Vanillic acid
13	Gallic acid
14	Ascorbic acid
15	Aesculetin
16	Caffeic Acid
17	Ferulic acid
18	Caffeine

19	Gallic Acid Ethyl Ester
20	Syringic acid
21	Resveratrol
22	3-Oxocostusic acid
23	Daidzein
24	Adenosine
25	Formononetin
26	Apigenin
27	Genistein
28	Naringenin
29	Arbutin
30	Biochanin A
31	Kaempferol
32	Luteolin
33	Atropine
34	Catechin
35	Epicatechin
36	Hispidulin
37	Ladanetin
38	Ellagic acid
39	Quercetin
40	Kumatakenin
41	Galangustin
42	Isorhamnetin
43	Quinine
44	7,3'-Dimethoxy-5,6,4'-trihydroxyisoflavone
45	Quercetin 3,3'-dimethyl ether
46	Gibberellic acid
47	Chlorogenic acid
48	Rosmarinic Acid
49	Ajugoside
50	Colchicine
51	8-Hydroxyquinoline sulfate monohydrate
52	stigmasterol
53	beta-Sitosterol
54	Vitexin
55	Isoorientin
56	Oxytetracycline hydrochloride
57	Hyperoside
58	Temephos

59	Salbutamol Sulphate
60	Naringin
61	Rutin
62	Hesperidin
63	Acetoside
